# Maternal Serum Levels of TNF-Alpha and IL-6 Long after Delivery in Preeclamptic and Normotensive Pregnant Women

**DOI:** 10.1155/2010/908649

**Published:** 2010-12-28

**Authors:** N. Vitoratos, E. Economou, C. Iavazzo, K. Panoulis, G. Creatsas

**Affiliations:** ^1^2nd Department of Obstetrics and Gynecology, Aretaieio Hospital, University of Athens, Athens, Greece; ^2^38, Seizani Str., Nea Ionia, 14231 Athens, Greece

## Abstract

*Aim*. To evaluate maternal TNF-alpha and IL-6 plasma levels in normotensive pregnant women, women with preeclampsia, and to examine the temporal changes in their levels from theantepartum to the postpartum period correlated with the regression of preeclampsia. *Method*. A prospective study was performed in the 2nd Department of Obstetrics and Gynecology, University of Athens. Blood samples were obtained: (1) antepartum at the time of clinical diagnosis of the syndrome, 2. 12-14 weeks postpartum. *Results*. No statistically significant differences were found in IL-6 levels, whereas a difference was found in TNF-alpha levels between preeclamptic and controls in antepartum period (0.80 pg/ml versus 0.60 pg/ml, *P* : .04). Long after delivery, TNF-alpha levels were significantly higher in preeclamptic compared to normotensive controls (0.86 pg/ml versus 0.60 pg/ml, *P* : .004). No difference was observed in TNF-alpha before and after delivery in both groups. No difference was noticed in IL-6 levels in women of normotensive group long after delivery compared to that before delivery. Long after delivery IL-6 levels were statistically significant higher in preeclamptic women compared to normal controls (3.53 ± 0.52 pg/ml versus 1.69 ± 0.48 pg/ml, *P* : .02). *Conclusion*. Preeclamptic women remain under a status of increased inflammatory stress up to 12-14 weeks postpartum despite the fact that all the other signs of preeclampsia are resolved.

## 1. Introduction

Although there is no systemic inflammation during pregnancy, circulating cytokines are found to be elevated in maternal plasma [1]. More specifically, normal pregnancy is characterized by local inflammatory response which leads to local production of proinflammatory cytokines that could be found in the systematic circulation. The presence of such cytokines in the systematic circulation might secondarily lead to subclinical systemic inflammatory response. In preeclampsia, a similar but exaggerated response occurs [2]. Preeclampsia is a severe pregnancy-specific disorder with an incidence which has been reported to be approximately 5–8% [3]. It is a syndrome defined by hypertension and proteinuria that also may be associated with myriad other signs and symptoms and often with subnormal fetal growth [4, 5]. The mechanisms responsible for the pathogenesis of preeclampsia have not yet been clearly identified, but reduced uterine perfusion and placental ischemia are an important initiating event in this disorder, and inflammatory cytokines are thought to link placental ischemia with cardiovascular and renal dysfunction symptoms seen in this disorder [6].

Maternal serum levels of IL-6 and TNF-alpha play a significant role in pathogenesis of preeclampsia [7]. TNF-alpha is produced by monocytes, induces apoptosis, and inhibits proliferation of trophoblast cells in preeclampsia [8]. The fact that higher circulating levels of TNF-alpha were observed in preeclampsia than in gestational hypertension suggests an association with disease severity [9]. Moreover, significantly increased soluble TNF-alpha receptors are found in the plasma of patients with preeclampsia [10]. Soluble TNF-alpha receptors bind with circulating TNF-alpha leading to a decrease of the ligand's availability [10]. At least two different views on the role of soluble receptors of TNF-a in systemic circulation have been expressed in the literature: (a) increased levels of TNF-*α* soluble receptors in systemic circulation may indicate either increased expression of the receptors on the cells' surface, and therefore the increased sensitivity and responsiveness of these cells to elevated levels of TNF-a, as it has been shown in the preeclamptic women of our study or increased presence of systemic circulation proteases of the extracellular part of transmembrane TNF-*α* receptor and (b) the presence of TNF-*α* soluble receptor in the systemic circulation which does not document their ability to bind TNF-*α* in systemic circulation, as the decay of the proteases is likely to affect the binding capacity. Furthermore, the poorly perfused and hypoxic placenta is thought to synthesize increased vasoactive factors, for example, soluble fms-like tyrosine kinase-1, cytokines (TNF-alpha, IL-6), and angiotensin II type 1 receptor antibodies) [11]. Such elevations could lead to endothelial dysfunction by decreasing bioavailable nitric oxide and increasing reactive oxygen species and endothelin-1 which in turn leads to altered renal function, decreased renal pressure natriuresis, increased total peripheral resistance, and hypertension [11, 12]. Ischemic placenta leads to endothelial cell activation/dysfunction and enhances TNF-alpha synthesis which leads to induced structural and functional alterations in endothelial cells leading to preeclampsia via transcriptional regulation of the endothelin-1 gene by TNF-alpha [13–17].

Conflicting results, however, are found regarding the role of circulating IL-6 and TNF-alpha in a review of the current literature. Many authors believe that circulating levels of TNF-alpha and IL-6 are enhanced in preeclamptic patients compared with normotensive and nonpregnant women [18]. On the contrary, others did not manage to identify a correlation between maternal serum levels of IL-6 or TNF-alpha and preeclampsia [19, 20]. Furthermore, there is no information about maternal blood concentrations of IL-6 and TNF-alpha in women with preeclampsia long time after delivery. 

In the present study, we have evaluated maternal TNF-alpha and IL-6 plasma levels in normotensive pregnant women as well as in women with preeclampsia. We have also examined whether the temporal changes in plasma TNF-alpha and IL-6 levels from the antepartum to the postpartum period correlate with the regression of preeclampsia.

## 2. Materials and Methods

Two groups of patients were studied: normal controls and women with preeclampsia. The preeclamptic group consisted of 17 normotensive women who later in their pregnancy developed preeclampsia. The diagnosis of preeclampsia was established according to the criteria developed by the National High Blood Pressure Education Programme Working Group [21]: systolic blood pressure of more than 140 mm Hg, diastolic pressure higher than 90 mm Hg on two different occasions after the 20th week of pregnancy and proteinuria defined as urine secretion of 0.3 gr of protein or higher in a 24-hour urine specimen. Blood pressure was measured with the blood pressure cuff placed on the left arm at heart level in sitting position.

All women included in preeclamptic group had severe preeclampsia because they met one or more of the following criteria: systolic blood pressure >160 mm Hg, diastolic blood pressure >100 mg Hg, proteinuria >2 grams in a 24-hour urine collection, blurred vision, epigastric or upper quadrant abdominal pain, vomiting, or subnormal fetal growth. The control group consisted initially of 19 gestation-age-matched normotensive pregnant women. Three women were excluded from the study because one developed gestational hypertension and two gestational diabetes later in pregnancy. The rest of the women (16 cases) remained normotensive and without any complication throughout their pregnancy. 

All women had similar socioeconomic status and were nonsmokers. None was a permanent drug user or had any known metabolic or renal disease. All women had been receiving ferrous sulfate supplementation during pregnancy and had hemoglobin levels of more than 10.5 gr/dl.

An informed consent was obtained from all women participating in the study. The study was approved by the ethics committee of our institution.

### 2.1. Procedure Blood Samples

Blood samples were obtained in two periods: (1) antepartum at the time of clinical diagnosis of the syndrome and (2) 12–14 weeks postpartum in normal women as well as in women with preeclampsia at a time when the harmful effects of preeclampsia were expected to be absent. Blood samples (15 ml) were collected into citrated vacutainers. The tubes remained for 30 minutes at 4°C, and the blood samples were centrifuged at 1600 g for 15 minutes at a temperature of 4°C. The supernatant plasma was separated and stored at −70°C. 

Enzyme-linked immunosorbent assay was used for quantitative detection of human TNF-alpha, Bender Medsystems GmbH (BMS223HS), Austria. The limit of detection of TNF-alpha was determined to be 0.13 pg/ml. The overall intraassay coefficient of variation has been calculated to be 8.5% while the overall interassay coefficient of variation has been calculated to be 9.8%. Enzyme-linked immunosorbent assay was used for quantitative detection of soluble human IL-6, Bender Medsystems GmbH (BMS213HS), Austria. The limit of detection of IL-6 was determined to be 0.02 pg/ml. The overall intraassay coefficient of variation has been calculated to be 6.9% while the overall interassay coefficient of variation has been calculated to be 8.0%.

### 2.2. Statistical Analysis

Statistical analysis was performed with a commercially available statistical package (SPSS v 17.0 Chicago IL). Normality of the pregnancy distribution of the studied variables was checked by Kolmogorov-Smirnov test. Student's *t*-test or paired *t*-test was used as appropriate for comparison of normally distributed variables, while nonparametric Wilcoxon rank-sum test was used for comparison of nonnormally distributed variables, and values are expressed as mean ± SE for normally distributed and as median  ±95 confidence intervals for nonnormally distributed variables. A *P* level of <.05 was considered significant.

## 3. Results

Demographic characteristics of the two groups are presented in Table 1. All women were Caucasian and married. There were no differences between the groups regarding maternal and gestational age at sampling. The mean systolic and diastolic blood pressure as well as the neonatal clinical characteristics is also presented.

Alterations in plasma IL-6 and TNF-alpha levels in antepartum (A) and postpartum (P) periods are shown in Table 2.

No statistically significant differences were found in plasma IL-6 levels between preeclamptic and controls (mean ± SE: 2.66 ± 1.37 pg/ml versus 2.44 ± 0.85 pg/ml, *P* : .9) in antepartum period. On the contrary, a statistically significant difference was found in TNF-alpha levels in women with preeclampsia compared to normal pregnant women (0.80 pg/ml range: 0.67–0.92 pg/ml versus 0.60 pg/ml range: 0.49–3.01 pg/ml, *P* : .04).

Long after delivery, TNF-alpha levels were significantly higher in preeclamptic women (mean ± SE: 0.86 pg/ml, range 0.78–0.94 pg/ml) compared to normotensive controls (mean ± SE: 0.60 pg/ml range: 0.50–3.87 pg/ml, *P* : .004). No difference was observed in maternal plasma TNF-alpha before and after delivery in both groups. A drop, but not a statistically significant one, was noticed in plasma IL-6 levels in women of normotensive group (mean ± SE: 1.69 ± 0.48 pg/ml) long after delivery compared to that (mean ± SE: 2.44 ± 0.85 pg/ml *P* : .4) before delivery.

An increase, but not a statistically significant one, was observed in plasma IL-6 levels of the preeclamptic women (mean ± SD: 3.53 ± 0.52 pg/ml) in postpartum period compared to that found before delivery (mean ± SD: 2.66 ± 1.37 pg/ml, *P* : .3).

As shown in Table 2, long after delivery, IL-6 levels were statistically significantly higher in preeclamptic women compared to normal controls (mean ± SD: 3.53 ± 0.52 pg/ml versus 1.69 ± 0.48 pg/ml, *P* : .02).

## 4. Discussion

TNF-alpha is a polypeptide cytokine produced by monocytes and macrophages and has a potentially cytotoxic effect to trophoblasts as demonstrated by in vitro experiments [22]. TNF-alpha circulates throughout the body responding to stimuli (infections, agents, or tissue injury) activating neutrophils, altering the properties of vascular endothelial cells [23, 24]. It has been shown that it inhibits the outgrowth of human trophoblast, and the injection of TNF-alpha into pregnant mice results in the termination of pregnancy [25]. Elevated TNF-alpha levels may cause endothelial dysfunction both directly and indirectly. IL-6 is a multifunctional cytokine that regulates immune responses and acute phase reactions and may play a central role in host defence mechanisms [26]. 

Conflicting results are found regarding the role of IL-6 and TNF-alpha in a review of the current literature. Many believe that circulating levels of TNF-alpha and IL-6 are enhanced in preeclamptic patients compared with normotensive and nonpregnant women [18]. In two different studies, Page et al. [27] and Tosun et al. [7] have recently shown that maternal serum levels of IL-6 and TNF-alpha are significantly increased in preeclamptic patients rising in a way that higher levels are found in patients with severe compared to mild preeclampsia. Trying to explain such findings, Cachovic et al. [28] showed that the fractional secretion of TNF-alpha is significantly reduced in preeclamptic women, and for this reason they suggest that the decreased clearance and altered renal excretion of TNF-alpha may lead to preeclampsia. On the contrary, others did not manage to identify a correlation between maternal serum levels of IL-6 or TNF-alpha and preeclampsia [19, 20]. Furthermore, Montagnana et al. [29] based on their results concluded that preeclampsia screening based on cytokines such as IL-6 or TNF-alpha is not proposed.

According to our results, only the circulating levels of TNF-alpha and not those of IL-6 were enhanced in women with preeclampsia when compared to normotensive women. The source of excess TNF-alpha in preeclampsia remains unclear. The maintenance of elevated TNF-*α* levels compared to those of controls, both directly and in the long period of time after completion of preeclampsia, could lead to increased stimulation of monocytes and macrophages, causing overproduction of free radicals: O2 and HOCl (based the oxidative stress theory). Thus, maintaining high TNF-*α* levels in systemic circulation may be responsible for the stimulation or disruption of vascular endothelium in the distant time after the removal of the causing factor of preeclampsia which could be expressed by increased production in the systemic circulation of IL -6. In this way, the longer-term clinical effects of preeclampsia in coronary vessels could be justified, as recent literature argues that preeclampsia is often followed in the future by coronary heart disease and cardiovascular events. Histological observations in near term placental bed biopsies, although still conflicting, indicate higher local expression of these cytokines in preeclamptic patients. Decidual monocytes and macrophages are also a rich source of TNF-alpha. The elevated secretion of TNF-alpha by placental villous tissue in response to hypoxia causes a reduction of endothelial cell viability and up-regulates the expression of the adhesion molecule E-selectin by the endothelial cell [30]. It is possible that syncytiotrophoblast could synthesize and release excessive amounts of proinflammatory cytokines such as TNF-alpha or IL-6 [31], but an analysis of production from chorionic villous explants failed to show the expected increase in protein or mRNA for TNF-alpha, IL-6, IL-1alpha, and IL-1beta in tissue from preeclampsia compared to that from normal pregnancy [32]. Thus, there is no convincing evidence that the preeclampsia placenta disseminates inflammatory cytokines into the maternal circulation [33]. In parallel, in vitro in a dually perfused placental cotyledon, most of placental TNF-alpha was released to the maternal side confirming the importance of placentally produced inflammatory mediators in the induction of the maternal systemic changes [34]. Hypoxia increases TNF-alpha synthesis by placental villous tissue in vitro [35]. Additionally, chemicals which trigger placental oxidative stress also stimulate elevated TNF-alpha production by villous explants [30]. On the other hand, NO hypoxia-driven changes in IL-6 synthesis by placental explants were documented. These data contradict the finding that preeclampsia is associated with decreased placental IL-6 production [7, 32].

Our results showing no changes of maternal circulating TNF-alpha and a drop, but not a significant one, of IL-6 levels long after delivery suggest that the source of the excessive production of TNF-alpha in preeclampsia may be monocytes, neutrophils, and unlikely placenta itself. This suggestion is in accordance to that of other authors [36].

The similar concentrations of IL-6 in maternal circulations in both groups in antepartum period may indicate that the role of circulating IL-6 is limited in the pathogenesis of preeclampsia and it is opposite to the suggestions of Jirik et al. [37] that the increased TNF-alpha production affects the production of IL-6 and its receptor. On the contrary, the elevated maternal concentrations of TNF-alpha may be a part of the pathogenesis of preeclampsia. Many authors suggest that elevated circulating TNF-alpha levels may be involved in the pathogenesis of preeclampsia [14, 38–40].

An interesting finding of this study is that elevated circulating TNF-alpha and IL-6 levels were detected long after delivery in women with preeclampsia. It is well known that the primary biological activities of proinflammatory cytokines include inflammation and endothelial cell activation [40]. Our findings suggest that preeclamptic women remain under a status of increased inflammatory stress up to 12–14 weeks postpartum despite the fact that all the other signs of preeclampsia are resolved. However, a question could be raised whether increased TNF-alpha levels might be the consequence rather than the cause of the symptoms.

It is being increasingly recognized that women with a history of preeclampsia are at an increased risk for developing cardiovascular diseases [41, 42]. Several mediators of endothelial cell dysfunction such as oxidative stress, various inflammation factors, and leukocyte adhesion may contribute to endothelial dysfunction in both preeclampsia and coronary artery disease [43].

Our study shows that circulating TNF-alpha, but not IL-6, levels are higher in preeclamptic women—at the time symptoms are presented—than in healthy pregnant women in the same period. So we may hypothesize that increased inflammatory factors seen in our material after delivery are likely to play a role in the pathogenesis of cardiovascular diseases seen late in life among women with preeclampsia.

## Figures and Tables

**Table 1:** Demographic and clinical characteristics of women with normal pregnancy (NP) and pregnancy complicated by preeclampsia (PE). There were no differences between the two groups unless otherwise indicated.

	NP:16	PE:17
Maternal age (years)	33.57 ± 4.43	31.14 ± 5.24
Gestational age at sampling (mean ± SD)	34.14 ± 3.63	32.71 ± 3.04
BMI before birth	25 ± 4.04	28.96 ± 6.71
Systolic blood pressure before birth (mean ± SD)	117.6 ± 8.6	175.22 ± 12.7*
Diastolic blood pressure before birth (mean ± SD)	71.8 ± 7.6	110.7 ± 7.51**
Neonatal weight (gr)	3275.71 ± 333.61	1699.29 ± 625.3*
Primigravida (N) (%)	10 (62.5)	17 (100)
Admission to the ICU (N) (%)	—	5 (31.2)
IUGR neonates (N) (%)	—	6 (37.5)*

*P < .05 versus control.

**P < .001 versus control.

**Table 2:** Alterations in maternal plasma TNF-alpha and IL-6 levels in preeclamptic women (PE) and normal control (NP) during the antepartum (A) and 12–14 weeks postpartum (P).

(A)	NP: *n*:16	PE: *n*:17	*P*
IL-6, mean ± SE (pg/ml)	2.44 ± 0.85	2.66 ± 1.37	.9
TNF-alpha, median (95CI) (pg/ml)	0.60 (0.49–3.01)	0.80 (0.67–0.92)	.04
(P)IL-6, mean ± SE (pg/ml)	1.69 ± 0.48	3.53 ± 0.52	.02
TNF-alpha, median (95CI) (pg/ml)	0.60 (0.50–3.87)	0.86 (0.78–0.94)	.004
